# Case Report: Bilateral vision-threatening anterior segment injury following periocular cosmetic high-intensity focused ultrasound

**DOI:** 10.3389/fmed.2026.1891995

**Published:** 2026-10-21

**Authors:** Hai Xuan Ho, Thanh Ngoc Tran, Loan Thi Hoang, Nhu Thuy Pham, Thoa Thi Doan, Lan Huong Thi Tran, Hong Son Cung

**Affiliations:** 1 Vietnam National Eye Hospital, Hanoi, Vietnam; 2 Clinical Research and Innovation Unit, Cung Hong Son International Eye Hospital, Hanoi, Vietnam; 3 Department of Ophthalmology, Bach Mai Hospital, Hanoi, Vietnam

**Keywords:** anterior segment injury, cosmetic ocular complication, high-intensity focused ultrasound, iris stromal disruption, lens opacity, ocular hypertension, periocular HIFU, thermal lens injury

## Abstract

High-intensity focused ultrasound (HIFU) is widely used for noninvasive cosmetic rejuvenation, including periocular procedures. However, when performed near the globe, focused thermal energy may injure multiple anterior segment structures. We report a 38-year-old woman who developed acute bilateral ocular pain, redness, and blurred vision shortly after periocular cosmetic HIFU for eyelid fat reduction and facial rejuvenation. The right eye was more severely affected. At presentation, best-corrected visual acuity was counting fingers at 0.5 m in the right eye and 20/40 in the left eye, with intraocular pressures of 30 and 26 mmHg, respectively. Slit-lamp examination showed bilateral anterior uveitis, corneal edema, keratic precipitates, endothelial pigment deposits, diffuse iris stromal disruption with pigment dispersion, and focal lens opacities consistent with presumed thermal lens injury. After corneal edema improved, focal, tapered corneal opacities became visible, especially in the right eye. The cosmetic clinic reported use of a 1.5-mm focal-depth probe at 7.0 MHz with an energy setting of approximately 1 J/cm^2^; however, further procedural details were not available. The patient received topical and systemic anti-inflammatory treatments, intraocular pressure-lowering medications, hypertonic sodium chloride ointment, and supportive care. During follow-up, inflammation subsided and intraocular pressure stabilized without medication. At about 2 months, visual acuity was stable at 20/30 in the right eye and 20/25 in the left eye, although corneal, iris, and lens abnormalities remained. This case broadens the known range of cosmetic periocular HIFU-related ocular injuries by documenting a severe bilateral, multistructural anterior segment injury with acute ocular hypertension, diffuse iris stromal disruption, focal corneal opacities, and persistent focal lens opacities. It emphasizes the importance of conservative energy use during periocular procedures, ocular protection, trained operators, and prompt eye examinations if symptoms occur after the procedure.

## 1 Introduction

High-intensity focused ultrasound (HIFU) is a non-invasive aesthetic technique that focuses acoustic energy at specific tissue depths to produce localized thermal coagulation and tissue remodeling (1). It is increasingly used for periocular rejuvenation, including eyelid laxity and periorbital wrinkles. Because the eyelid is thin and the globe lies close to the treatment field, periocular use requires careful control of probe position, angulation, focal depth, energy and ocular protection (2, 3).

Ocular complications after periocular HIFU are uncommon but increasingly recognized. Reported injuries include corneal opacity, acute ocular hypertension, anterior uveitis with iris atrophy, conjunctival hemorrhage, and cataract formation (3–8). Most reported cases have involved unilateral or localized anterior segment damage. However, severe bilateral injury with diffuse iris stromal disruption, prominent pigment dispersion, acute ocular hypertension, corneal opacities, and focal lens opacities remains rarely described. Herein, we report a case of severe bilateral anterior segment injury following periocular cosmetic HIFU in a 38-year-old woman. The case was characterized by acute ocular hypertension, corneal edema, focal corneal opacities, diffuse iris stromal disruption with pigment dispersion, anterior uveitis, and focal lens opacities consistent with presumed thermal injury. This case adds a bilateral, multistructural, vision-threatening clinical picture to the spectrum of ocular complications associated with cosmetic periocular HIFU.

## 2 Case report

A 38-year-old woman from a rural province was referred to Vietnam National Eye Hospital from a provincial eye hospital with a 3-day history of acute ocular pain, redness, and severe blurred vision in both eyes, more pronounced in the right eye. On Day 0, she underwent bilateral periocular high-intensity focused ultrasound (HIFU) treatment at a local private cosmetic clinic for eyelid fat reduction and facial rejuvenation. During treatment around the right eye, she experienced moderate, sharp ocular pain and informed the technician. The energy setting was reportedly reduced, after which the pain subsided. The same adjusted setting was then used for the left periocular region without immediate discomfort. Shortly after the procedure, she developed persistent dull ocular pain and blurred vision in both eyes, worse in the right eye. She was reassured that these symptoms were transient and likely related to temporary eyelid swelling and was advised to take analgesics and rest.

On Day 1, her symptoms persisted and worsened, with increased blurred vision, ocular pain, and redness. She sought urgent evaluation at a provincial eye hospital, where she was diagnosed with bilateral elevated intraocular pressure (IOP) and corneal edema. Initial treatment included Maxitrol® ophthalmic suspension (dexamethasone 0.1% w/v, neomycin sulfate 3,500 IU/mL, and polymyxin B sulfate 6,000 IU/mL) four times daily, Nevanac® (nepafenac 0.1% w/v) twice daily, and Combigan® (brimonidine tartrate 0.2% w/v, timolol 0.5% w/v) twice daily. She was subsequently referred to our tertiary center at her request.

At our center, the patient denied prior similar ocular symptoms and reported no known systemic or ocular disease. We contacted the cosmetic clinic to obtain procedural details. The clinic only reported that a BICO HIFU Double S device was used with a 1.5-mm focal-depth probe operating at 7.0 MHz, an energy setting of approximately 1 J/cm^2^, adjustable in increments of 0.05 J/cm^2^. No further details, including exact treatment sites, the number of shots, or safety precautions, were provided.

At her first presentation to our center on Day 3, best-corrected visual acuity (BCVA) was counting fingers at 0.5 m in the right eye and 20/40 in the left eye. Despite ongoing treatment with topical brimonidine/timolol twice daily, IOP remained elevated at 30 mmHg in the right eye and 26 mmHg in the left eye, measured using a non-contact tonometer (TONOPACHY^TM^ NT-530P; NIDEK CO., LTD., Gamagori, Aichi, Japan). No relative afferent pupillary defect was detected. Anterior segment examination of the right eye showed ciliary injection, marked corneal edema, scattered small-to-medium keratic precipitates, and pigment deposits on the corneal endothelium. The anterior chamber showed 1+ cells and 2+ flare according to the SUN grading scheme (9). The iris was diffusely damaged, with stromal disruption and marked pigment dispersion into the anterior chamber. No posterior synechiae were observed (Figure 1A). After pharmacologic dilation with Mydrin-P® (tropicamide 0.5% and phenylephrine hydrochloride 0.5%), the pupil appeared irregular and distorted. The lens showed multiple focal opacities with a tapered, cone-shaped configuration compatible with focal thermal injury. Vitreous and fundus examination was limited by corneal edema and anterior segment inflammation, but no definite posterior segment abnormality was identified.

**Figure 1:**
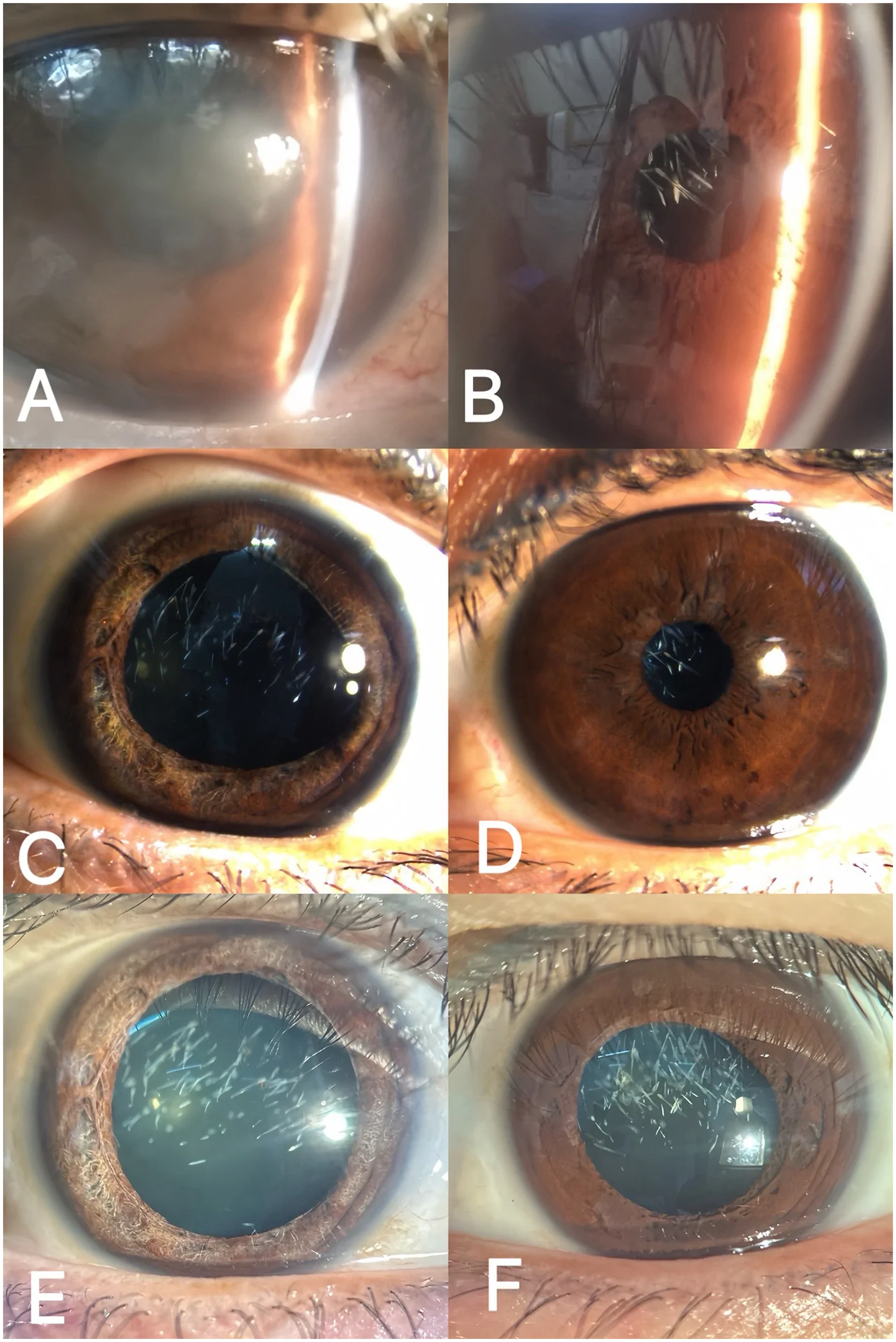
Serial slit-lamp photographs showing the evolution of bilateral anterior segment injury after periocular cosmetic high-intensity focused ultrasound. **(A,B)** Initial presentation before pharmacologic dilation, showing more severe anterior segment involvement in the right eye than in the left eye. **(C,D)** Day 23 after cosmetic HIFU, after improvement of corneal edema and anterior chamber inflammation. The right eye **(C)** was photographed after pharmacologic dilation, whereas the left eye **(D)** was photographed without dilation. **(E,F)** Final follow-up photographs obtained after pharmacologic dilation of both eyes, showing stable residual anterior segment changes. Right eye: A, C, E. Left eye: B, D, F.

In the left eye, there was mild ciliary injection with minimal corneal edema. A few small keratic precipitates and pigment deposits were noted on the corneal endothelium. The anterior chamber showed 0.5+ cells and 1+ flare. The iris also showed stromal injury and pigment dispersion, although less extensive than in the right eye, with focal irregular areas and an irregular pupil after pharmacologic dilation. No posterior synechiae were observed. Similar focal lens opacities were present, although less extensive than in the right eye (Figure 1B). Vitreous and fundus examination was unremarkable.

B-scan ultrasonography revealed scattered vitreous opacities without evidence of retinal detachment, choroidal abnormality, or other remarkable posterior segment findings. In the absence of corresponding posterior segment inflammatory findings, these echoes were nonspecific and not clearly attributable to HIFU-related vitreous inflammation. Optical coherence tomography (OCT) of the right eye was limited by marked corneal edema and media opacity. In the left eye, OCT findings were unremarkable.

Given the clinical presentation and the close temporal relationship to periocular HIFU exposure, the findings were considered consistent with HIFU-associated thermal injury, with bilateral acute anterior uveitis, elevated intraocular pressure, corneal edema, iris stromal injury, and focal lens opacities. The clinical course, key examination findings, and treatment are summarized in Table 1. Intraocular pressure remained inadequately controlled with topical brimonidine/timolol. The previously prescribed topical medications were continued at the same frequencies. Hypertonic sodium chloride 6% ointment was prescribed three times daily for the right eye to facilitate the resolution of corneal edema. Preservative-free sodium hyaluronate 0.15% w/v was added bilaterally. Pirenoxine 0.005% w/v ophthalmic suspension was empirically prescribed four times daily as supportive therapy for the focal lens opacities. Oral acetazolamide 250 mg twice daily was initiated for intraocular pressure control.

**Table 1:** Timeline of clinical findings and management following the cosmetic HIFU procedure.

Time point	BCVA, right/left eye	IOP, right/left eye	Key clinical findings	Treatment and clinical course
Day 0	Not assessed	Not assessed	Bilateral periocular cosmetic HIFU. Moderate sharp ocular pain occurred during right periocular treatment; energy setting was reportedly reduced. Shortly after treatment, persistent bilateral ocular pain and blurred vision developed, worse in the right eye.	The patient was initially reassured that the symptoms were transient and was advised to take analgesics and rest.
Day 1	Not available	Not available	Worsening bilateral ocular pain, redness, and blurred vision. At a provincial eye hospital, bilateral elevated IOP and corneal edema were diagnosed.	Topical dexamethasone/neomycin/polymyxin B four times daily, nepafenac twice daily, and brimonidine/timolol twice daily were initiated.
Day 3 First presentation at our center	CF at 0.5 m/20/40	30/26 mmHg	Bilateral anterior uveitis, corneal edema, keratic precipitates, endothelial pigment deposits, pigment dispersion, iris stromal injury, and focal lens opacities consistent with presumed thermal lens injury. Right eye more severely affected. B-scan showed scattered nonspecific vitreous opacities without retinal detachment or choroidal abnormality.	Provincial-hospital topical therapy was continued. Added hypertonic sodium chloride 6% ointment for the right eye, together with preservative-free artificial tears, pirenoxine, and oral acetazolamide 250 mg twice daily.
Day 6	CF at 2 m/20/30	17/16 mmHg	Symptoms improved. Right corneal edema and ciliary injection partially improved; AC inflammation persisted. Iris and lens findings stable.	Added oral methylprednisolone at a prednisolone-equivalent dose of 20 mg/day. Continued acetazolamide for 5 more days and maintained topical treatment.
Day 13	20/50/20/30	21/18 mmHg	Further improvement. Right corneal edema markedly improved. No AC cells. Residual flare and floating pigment remained. Iris and lens findings stable.	Discontinued hypertonic ointment. Tapered oral methylprednisolone to 10 mg/day prednisolone-equivalent dose. Tapered topical corticosteroid; continued nepafenac, artificial tears, pirenoxine, and brimonidine/timolol.
Day 23	20/30/20/30	21/20 mmHg	Inflammation controlled. After corneal edema resolved, focal tapered corneal opacities became apparent, more prominent in the right eye. Iris and lens findings stable.	Tapered oral methylprednisolone to 5 mg/day prednisolone-equivalent dose. Reduced topical corticosteroid to once daily. Continued nepafenac, artificial tears, and pirenoxine. Brimonidine/timolol discontinued; IOP reassessment planned.
Day 33	20/30/20/25	20/20 mmHg	No active AC inflammation. Corneal opacities, iris damage, and lens opacities remained stable. IOP remained controlled without pressure-lowering medication.	Oral and topical corticosteroids discontinued. Nepafenac continued for 2 more weeks, then stopped. Artificial tears and pirenoxine continued.
Approximately Day 63	20/30/20/25	19/19 mmHg	No recurrence of inflammation. Persistent right iris stromal disruption, irregular pupil, focal corneal opacities, and lens opacities. Left eye remained quiet and stable.	Observation continued. Cataract surgery deferred because visual acuity was functionally satisfactory and stable. Patient advised local follow-up and avoidance of further periocular HIFU.

AC, anterior chamber; BCVA, best-corrected visual acuity; CF, counting fingers; HIFU, high-intensity focused ultrasound; IOP, intraocular pressure.

On Day 6 after HIFU, ocular discomfort had improved. BCVA increased to counting fingers at 2 m in the right eye and 20/30 in the left eye. IOP decreased to 17 mmHg in the right eye and 16 mmHg in the left eye. Anterior segment examination showed gradual improvement in ciliary injection and corneal edema in the right eye, with 1+ anterior chamber cells and 1+ flare. Iris damage and focal lens opacities remained unchanged. As media clarity improved, vitreous and fundus examination revealed no remarkable posterior segment findings. In the left eye, inflammation also improved, with stable iris and lens findings.

Given the partial response to topical anti-inflammatory treatment, oral methylprednisolone at a prednisolone-equivalent dose of 20 mg/day was added. Oral acetazolamide was continued for 5 additional days, and topical brimonidine/timolol and the remaining topical medications were continued. Given the patient's geographic constraints, follow-up was scheduled, and she was instructed to seek urgent local care if pain, redness, or blurred vision worsened before the scheduled visit.

On Day 13 after HIFU, symptoms had further improved. BCVA was 20/50 in the right eye and 20/30 in the left eye. IOP was 21 mmHg and 18 mmHg, respectively. Right corneal edema and ciliary injection had markedly improved. The anterior chamber showed no cells, 1+ flare, and residual floating pigment. Iris damage and focal lens opacities were stable in both eyes. Hypertonic sodium chloride ointment was discontinued. Oral methylprednisolone was tapered to a prednisolone-equivalent dose of 10 mg/day. Topical dexamethasone/neomycin/polymyxin B was tapered, and nepafenac, artificial tears, and pirenoxine were continued. IOP remained controlled with topical brimonidine/timolol.

On Day 23 after HIFU, BCVA improved to 20/30 in both eyes. IOP was 21 mmHg in the right eye and 20 mmHg in the left eye without systemic acetazolamide. Anterior segment inflammation remained controlled, with no cells and only mild residual flare and floating pigment. After resolution of corneal edema, multiple focal corneal opacities with a tapered configuration became apparent in the right eye, predominantly involving the central cornea, with pigment deposits on the posterior corneal surface (Figure 2C). Similar but much subtler changes were also observed in the left eye. These findings were not clearly appreciated at earlier examinations due to overlying corneal edema, particularly in the more severely affected right eye. Scheimpflug imaging using the Pentacam® HR (OCULUS Optikgeräte GmbH, Wetzlar, Germany) demonstrated an open anterior chamber configuration and focal lens opacities in both eyes. The selected meridional scans were 172^o^ in the right eye and 188^o^ in the left eye, chosen because they provided the clearest visualization of the deepest visible lens opacities. On these sections, the anterior chamber angles measured 40.2^°^ in the right eye and 37.0^0^ in the left eye, without apparent angle closure. Along the same measured axes, the distances from the corneal apex to the deepest visible lens opacities were approximately 6.14 mm and 5.70 mm in the right and left eyes, respectively (Figures 2A,B). Iris damage and focal lens opacities remained stable in both eyes (Figures 1C,D). Oral methylprednisolone was tapered to a prednisolone-equivalent dose of 5 mg/day. Topical corticosteroids were reduced to once daily, and nepafenac, artificial tears, and pirenoxine were continued. Topical brimonidine/timolol was discontinued to assess IOP without pressure-lowering medication.

**Figure 2:**
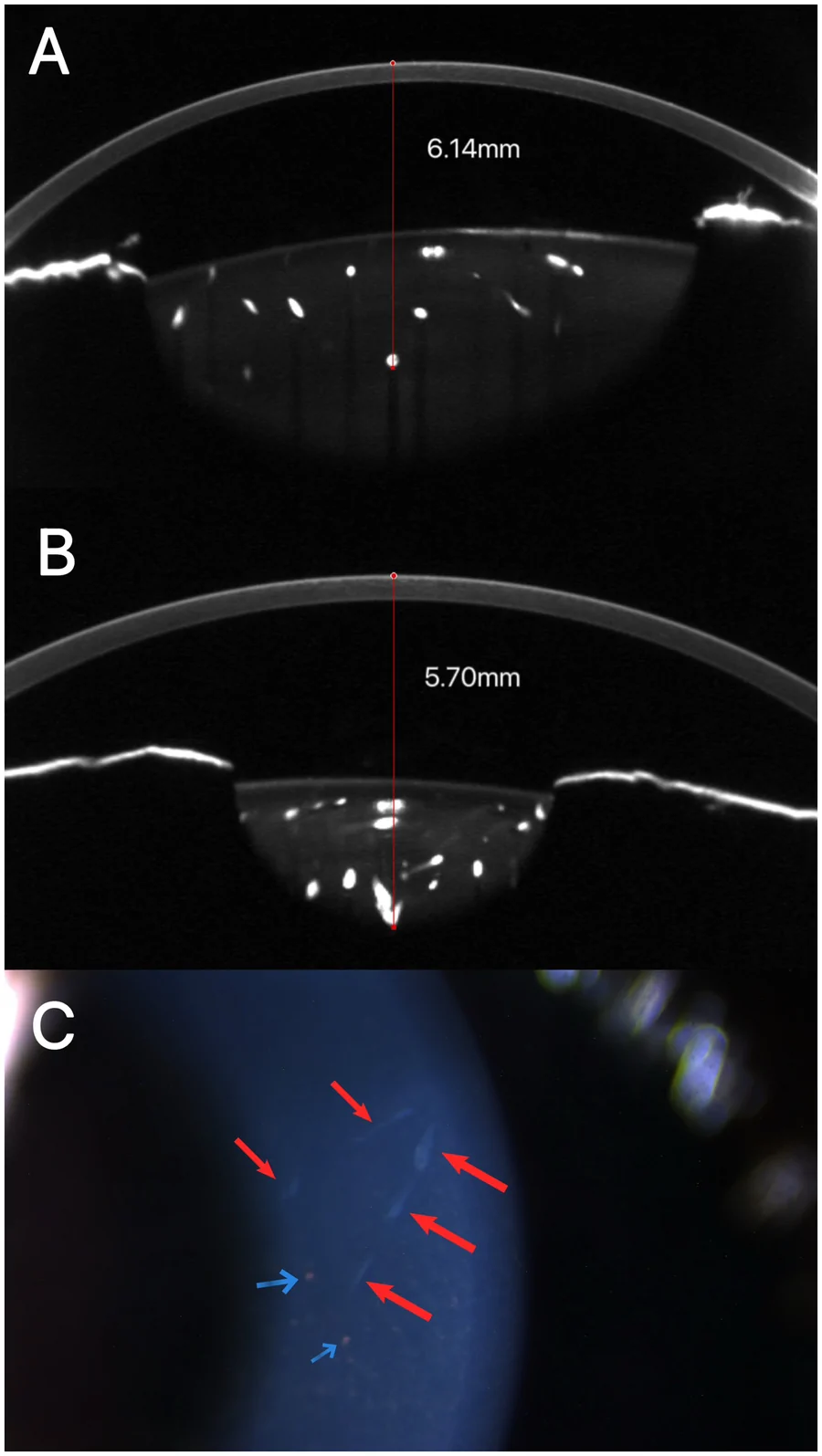
Scheimpflug and slit-lamp findings on Day 23 after cosmetic HIFU. **(A)** Right-eye Scheimpflug image at the 172o meridian and **(B)** left-eye image at 188o, selected for optimal visualization of the deepest visible focal lens opacities. Both eyes showed an open anterior chamber configuration without apparent angle closure. **(C)** High-magnification slit-lamp photograph of the right eye showing multiple focal tapered corneal opacities (red arrows) and pigment deposits on the posterior corneal surface (blue arrows).

On Day 33 after HIFU, BCVA improved to 20/30 in the right eye and 20/25 in the left eye. IOP was 20 mmHg in both eyes without pressure-lowering medication. No active anterior chamber inflammation was observed. The focal corneal opacities remained stable, with greater severity in the right eye. Iris damage and focal lens opacities also remained stable. Oral and topical corticosteroids had been discontinued. Topical nepafenac was continued for 2 additional weeks and then stopped. Artificial tears and pirenoxine were continued as adjunctive therapy. Further follow-up was scheduled, and the patient was advised to seek care at a local eye hospital if ocular pain, redness, recurrent blurred vision, or other concerning symptoms developed.

At approximately Day 63 after HIFU, BCVA remained stable at 20/30 in the right eye and 20/25 in the left eye. IOP was 19 mmHg in both eyes. There was no recurrence of inflammation. The right iris remained diffusely damaged with stromal disruption, and the pupil remained irregular after pharmacologic dilation. The focal corneal opacities and lens opacities were unchanged (Figure 1E). No posterior synechiae or posterior segment abnormalities were noted. The left eye remained quiet, with stable cornea, iris, and lens findings (Figure 1F). Because visual acuity remained functionally satisfactory and stable despite persistent focal lens opacities, cataract surgery was deferred, and the patient was managed conservatively with observation.

As inflammation had resolved and all findings were stable, the patient was advised to continue observation at a local eye center due to geographic constraints. She was advised to wear sunglasses to reduce glare or photophobia, to avoid further periocular HIFU treatment, and to seek prompt eye care if she experienced pain, redness, blurred vision, halos, or signs of recurrent inflammation or increased IOP. At the time of manuscript preparation, she reported that her ocular condition had remained stable.

## 3 Discussion

HIFU is increasingly used in aesthetic medicine to deliver focused thermal energy at selected tissue depths, producing tissue contraction and remodeling with visible cosmetic effects, limited treatment sessions, and little recovery time (1, 10). The periocular region leaves little margin for error because the eyelid is thin and the globe lies immediately beneath the treatment field. Inadequate probe position, angulation, focal depth, energy setting, or ocular protection may therefore expose the eye to injury (2, 5). Previous reports have described corneal opacity, anterior uveitis, iris atrophy, cataract formation, and occasional IOP elevation after periocular HIFU (3–8). Our case adds a severe bilateral, multistructural phenotype with acute ocular hypertension, diffuse iris stromal disruption, focal corneal opacities, and persistent lens opacities.

Focused ultrasound creates thermal coagulation points at predetermined depths, where temperatures may reach 65–75 °C and induce protein denaturation, collagen contraction, and subsequent wound-healing responses (10). This effect is desirable when confined to dermal or subdermal targets. If energy is transmitted toward the globe, ocular tissues may be injured. In our patient, the tapered corneal and lens opacities, diffuse iris damage, and pigment dispersion were compatible with focal thermal injury. Corneal stromal collagen and lens proteins may undergo thermal denaturation, while iris tissue may be disrupted by localized energy exposure. The corneal opacities became clearer only after edema resolved, particularly in the more severely affected right eye, suggesting that they had been obscured during the acute phase rather than developing later.

Slit-lamp and Scheimpflug imaging showed focal, tapered, cone-shaped lens opacities compatible with thermal injury. Although this pattern suggests injury along a focused energy pathway, the three-dimensional distribution and relationship to the true acoustic focal zone could not be determined. We therefore describe the finding broadly as HIFU-associated thermal lens injury rather than assigning it to a specific cortical layer. No retinal or choroidal abnormalities were identified, suggesting predominantly anterior segment injury, although longer follow-up is needed.

An additional consideration is whether the focal-depth probe reported by the cosmetic clinic accurately reflected the probe used during treatment. The clinic reported use of a BICO HIFU Double S device with a 1.5-mm focal-depth probe but no treatment record, probe photograph, device documentation, or other procedural information was available for independent verification. Similar HIFU systems are available with probes designed for different nominal focal depths, including 1.5, 3.0, and 4.5 mm (10). Therefore, inadvertent selection of a deeper focal-depth probe cannot be excluded. On Scheimpflug imaging, the deepest visible lens opacities were approximately 6.14 mm and 5.70 mm posterior to the corneal apex in the right and left eyes, respectively. These measurements represent the deepest visible extent of the opacities on selected Scheimpflug meridians and should not be interpreted as direct estimates of HIFU focal depth. The treatment angle and map, eyelid thickness and compression, exact probe position, and the finite spatial extent of thermal injury around the focal zone may all have influenced the observed distribution of lens opacities. Accordingly, inadvertent use of a deeper focal-depth probe remains a plausible but unproven explanation for the injury pattern. This uncertainty has important practical safety implications, supporting standardized pre-procedure verification of probe type, focal depth, energy setting, and treatment area. Manufacturers may further reduce selection errors through clearer probe differentiation, such as distinct labeling, color coding, or automatic device recognition of probe depth.

Safe periocular HIFU parameters have not been standardized (1). Uneventful treatment generally used relatively low nominal energy settings (approximately 0.2–0.5 J) with ocular shielding and careful probe handling (2, 11). Reported injuries ranged from anterior chamber inflammation, iris damage, and acute ocular hypertension to visually significant lens opacification requiring cataract surgery (Table 2) (3–8). Xiao et al. reported ocular injury at 0.9 J/cm^2^ (5); in our patient, the clinic reported using a 1.5-mm focal-depth probe operating at 7.0 MHz and approximately 1 J/cm^2^, adjustable in 0.05 J/cm^2^ increments. Because treatment devices, energy metrics, focal depths, and procedural protocols differ across reports, these values are not directly comparable and should not be interpreted as defining a specific injury threshold but rather as a reminder to choose treatment parameters cautiously when working in the delicate periocular region. The treatment map, number of shots, probe angulation, operator qualification, and ocular shielding in our patient were unavailable. Nevertheless, the close temporal relationship and multistructural pattern were consistent with unintended ocular exposure during periocular treatment.

**Table 2:** Comparison of previously reported ocular injuries following periocular cosmetic focused ultrasound.

Study	Laterality	Reported treatment parameters/ocular protection	Main ocular findings	IOP (mmHg)	Management	Outcome
Chen et al., 2018 (8)	Unilateral, OD	Energy/device parameters not reported. Ocular protection not reported.	Anterior chamber inflammation, iris pigment detachment, accommodative spasm/myopia, RAPD and pericentral scotoma.	31 (OD) 16 (OS)	Anti-glaucoma treatment; later cycloplegia and accommodative training.	IOP decreased to 21 mmHg after 1 day. Iris damage persisted; accommodative symptoms improved with treatment.
Roditi et al., 2020 (7)	Unilateral, OD	Parameters not reported. No protective eye gear.	Four drop-like dense lens opacities with stellate posterior capsular opacity; rapid progression. Corneal, anterior chamber, and iris initially normal.	Normal	Phacoemulsification + IOL implantation	UDVA improved from 20/160 to 20/25 and CDVA was 20/15 at 1 month postoperative.
Ikoma et al., 2022 (6)	Unilateral, OS	Irradiation dose unknown. No protective eye shield.	Multiple drop-like lens opacities extending toward posterior capsule with rosette-like posterior subcapsular opacity. No corneal or iris abnormality.	Normal	Cataract surgery with IOL implantation	Vision remained severely reduced at 20/630 before surgery. Postoperative CDVA improved to 20/20.
Marafon et al., 2023 (3)	Unilateral, OD	Ultraformer; detailed probe/power settings unavailable. No eye protection.	Three punctiform corneal epithelial defects with stromal edema/infiltration and severe anterior uveitis; later residual full-thickness stromal opacities. Iris atrophy and peripheral anterior cortical lens opacities.	16	Topical prednisolone, tapered over 30 days	Inflammation resolved. At 6 months, VA remained 20/20 despite residual corneal opacities, iris atrophy, and lens opacity. No surgery required.
Xiao et al., 2024 (5)	Bilateral lens involvement. OS more severe.	SHURINK-A; 0.9 J/cm^2^, focal depth 2.0 mm. Ocular protection not reported.	Bilateral streak/tadpole-shaped lens opacities; OS iridocyclitis, KPs, pigment dispersion/iris depigmentation, conjunctival hemorrhage and capsular changes.	Normal (OU)	Topical prednisolone, pranoprofen, and levofloxacin.	Inflammation resolved rapidly, but lens opacities persisted. At 1 year, the left lens opacity had progressed and VA was 20/25 OS.
Makibara et al., 2025 (4)	Unilateral, OD	Device and treatment parameters unavailable. Patient was not instructed to use eye protection.	Corneal edema, temporal iris atrophy, and acute lens opacity with three linear fibrotic points extending from posterior to anterior capsule.	10 (OU)	Phacoemulsification + IOL implantation.	Postoperative CDVA improved to 20/20 by day 14.
Present case	Bilateral, OD > OS	BICO HIFU Double S; 1.5-mm focal-depth probe, 7.0 MHz, ∼1 J/cm^2^, adjustable in increments of 0.05 J/cm^2^, were reported. Information on ocular protection unavailable.	Bilateral corneal edema followed by focal opacities, anterior uveitis, diffuse iris stromal disruption with pigment dispersion, and focal lens opacities	30 (OD) 26 (OS)	Topical and systemic anti-inflammatory therapy plus systemic/topical IOP-lowering treatment. Conservative management of lens opacities.	At ∼2 months, VA was 20/30 OD and 20/25 OS. IOP remained stable without medication. Inflammation resolved but corneal, iris, and lens abnormalities persisted.

UDVA, uncorrected distance visual acuity; CDVA, corrected distance visual acuity; HIFU, high-intensity focused ultrasound; IOP, intraocular pressure; IOL, intraocular lens; OD, right eye; OS, left eye; OU, both eyes; KPs, keratic precipitates; RAPD, relative afferent pupillary defect; VA, visual acuity. Device terminology, treatment parameters, and energy units are presented as reported in the original publications and should not be interpreted as directly comparable across different HIFU systems.

Our patient required broader treatment because anterior chamber inflammation, acute ocular hypertension, corneal injury, diffuse iris stromal disruption, and focal lens opacities occurred together bilaterally. Topical anti-inflammatory therapy was continued, systemic methylprednisolone was added when inflammation improved only partially, and intraocular pressure was controlled with oral acetazolamide and topical brimonidine/timolol during the acute phase. Once inflammation subsided and intraocular pressure stabilized, corticosteroids were tapered and pressure-lowering medications were discontinued. Nepafenac was continued for a further two weeks as adjunctive anti-inflammatory therapy during the step-down phase and was then discontinued. No corneal epithelial defect was documented during treatment.

Pirenoxine was used empirically as supportive therapy for thermal lens injury. Experimental studies suggest that pirenoxine may reduce ion-induced lens protein turbidity and may have antioxidative effects, particularly in cortical lens opacity (12). However, there is no established evidence that it can reverse thermal lens injury, and it was therefore not considered a primary treatment in this case. The favorable short-term course was more likely related to early recognition, control of inflammation, and timely reduction of intraocular pressure. Cataract surgery was deferred because visual acuity remained functionally satisfactory and stable.

This case has limitations. Procedural information from the cosmetic clinic was incomplete, and no pre-procedure ophthalmic examination or imaging was available for direct comparison. Gonioscopy and specular microscopy were not performed during the acute phase, limiting detailed assessment of the anterior chamber angle and corneal endothelial injury. Some clinical photographs also varied in quality because they were obtained during routine care. Nevertheless, serial imaging documented the main clinical course, including resolution of corneal edema and persistence of corneal, iris, and lens changes. Longer follow-up is still needed to determine whether the focal lens opacities, iris damage, or intraocular pressure remain stable over time.

Overall, this case illustrates that periocular HIFU injury may extend beyond transient inflammation to persistent multistructural anterior segment damage. These findings support the need for stricter periocular safety protocols and prompt ophthalmic assessment when ocular symptoms develop.

## 4 Conclusion

Cosmetic periocular HIFU may be associated with serious ocular injury involving multiple anterior segment structures. This injury involved more than temporary inflammation, including bilateral corneal edema and opacities, extensive iris stromal disruption, acute ocular hypertension, and thermal lens injury. Although the inflammation and intraocular pressure were controlled with medication in our patient, persistent structural changes remained at the last follow-up. When performing HIFU near the eye, it is crucial to use conservative energy levels, safeguard the eye, ensure the procedure is performed by trained professionals, and arrange prompt ophthalmic evaluation if ocular symptoms occur.

## Patient perspective

The patient expressed concern about the acute visual symptoms that occurred after the cosmetic procedure. After treatment, she was reassured by the improvement in symptoms and visual acuity, and she agreed to continue follow-up locally because of geographic constraints.

## Patient consent

Written informed consent was obtained from the patient for publication of clinical information and images.

## Authorship

All authors attest that they meet the current ICMJE criteria for Authorship.

## Data Availability

The original contributions presented in the study are included in the article/Supplementary Material, further inquiries can be directed to the corresponding author.

## References

[1] Haykal D Sattler S Verner I Madhumita M Cartier H. A systematic review of high-intensity focused ultrasound in skin tightening and body contouring. Aesthet Surg J. (2025) 45:690–8. 10.1093/asj/sjaf053 40184185

[2] Hwang Y Kim J-H Lee HE Jie IWK Song JK Jalali A. Efficacy of high-intensity focused ultrasound for treating upper and lower eyelid sagging. JPRAS Open. (2026) 48:147–54. 10.1016/j.jpra.2025.10.036 41438879 PMC12719753

[3] Marafon SB Marinho DR Kwitko S. Cornea opacity, uveitis with iris atrophy and lens damage following cosmetic high-intensity ultrasound of the eyelid: a case report. BMC Ophthalmol. (2023) 23:230. 10.1186/s12886-023-02947-9 37217891 PMC10201742

[4] Makibara RN de Oliveira LP Rocha MDdA. Unilateral acute cataract after facial aesthetic procedure: a case report. Arq Bras Oftalmol. (2025) 88:e2023–4. 10.5935/0004-2749.2023-0248 PMC12668691 39319911

[5] Xiao G Jan C Hong L Ye J Li N Li Y. Eye injuries caused by high intensity macro and micro focused ultrasound treatment: a case report. BMC Ophthalmol. (2024) 24:390. 10.1186/s12886-024-03645-w 39227863 PMC11370310

[6] Ikoma T Shibata T Shibata N Mito T Kubo E Sasaki H. Acute cataract by a high-intensity focused ultrasound procedure: a case report. BMC Ophthalmol. (2022) 22:164. 10.1186/s12886-022-02390-2 35397531 PMC8994384

[7] Roditi E Wasser LM Gelman E Zadok D. Cataract induced by eyelid cosmetic treatment with intense focused ultrasound. J Cataract Refract Surg. (2020) 46:e27–9. 10.1097/j.jcrs.0000000000000357 32818362

[8] Chen Y Shi Z Shen Y. Eye damage due to cosmetic ultrasound treatment: a case report. BMC Ophthalmol. (2018) 18:214. 10.1186/s12886-018-0891-2 30157786 PMC6114535

[9] Jabs DA Nussenblatt RB Rosenbaum JT, Standardization of Uveitis Nomenclature (SUN) Working Group. Standardization of uveitis nomenclature for reporting clinical data. Results of the first international workshop. Am J Ophthalmol (2005) 140:509–16. 10.1016/j.ajo.2005.03.057 16196117 PMC8935739

[10] Biskanaki F Tertipi N Sfyri E Kefala V Rallis E. Complications and risks of high-intensity focused ultrasound (HIFU) in esthetic procedures: a review. Appl Sci. (2025) 15:4958. 10.3390/app15094958

[11] Kim J Jung S. Efficacy of single dot ultrasound combined with radiofrequency for low eyelid laxity. J Cosmet Dermatol. (2023) 22:2139–40. 10.1111/jocd.15668 36755515

[12] Upaphong P Thonusin C Choovuthayakorn J Chattipakorn N Chattipakorn SC. The possible positive mechanisms of pirenoxine in cataract formation. Int J Mol Sci. (2022) 23:9431. 10.3390/ijms23169431 36012695 PMC9408903

